# Study on the adhesion performance of basalt fiber and polypropylene fiber based on unified phase-field theory and cohesive zone model

**DOI:** 10.1371/journal.pone.0347537

**Published:** 2026-05-04

**Authors:** Zhao Wen, Wang Simeng, Wang Huiming, Cai Guanghui

**Affiliations:** 1 College of Civil Engineering and Architecture, Xinjiang University, Urumqi, China; 2 Xinjiang Key Lab of Building Structure and Earthquake Resistance, Xinjiang, Urumqi, China; China Construction Fourth Engineering Division Corp. Ltd, CHINA

## Abstract

In recent years, mixing an appropriate amount of flexible fibers(e.g., basalt fibers, polypropylene fibers, etc.) into concrete has become a common practice. Numerous engineering applications have shown that adding one or more types of flexible fibers not only significantly enhances the mechanical properties of concrete, such as flexural and tensile strength, but also markedly improves its durability by resisting sulfate and chloride ion attack and reducing crack formation. Building upon this practical significance, this study developed finite element models to investigate the pull-out behavior of basalt fibers and polypropylene fibers based on the unified phase-field theory (UPFT) coupled with the cohesive zone model (CZM). The fiber-matrix interfacial adhesion was simulated using zero-thickness cohesive elements to investigate and analyze the adhesion performance of the two fiber types pulled out from the concrete matrix. A series of numerical simulations was conducted to evaluate the effects of embedment depth, fiber diameter, and interfacial properties on the mechanical response. The predicted pull-out loads exhibited good agreement with experimental results, confirming the reliability and accuracy of the proposed modeling framework. The findings indicate that the peak pull-out load of both basalt and polypropylene fibers increases with greater embedment depth and larger fiber diameter. Furthermore, enhancing either the interfacial adhesion strength or the matrix strength significantly improves the reinforcing effectiveness of the fibers within the concrete matrix.

## 1. Introduction

As a predominant construction material, concrete plays a crucial supporting role in various engineering projects such as bridges, buildings, ports, dams, and roads. However, concrete exhibits inherent defects, including low tensile strength, high self-weight, and susceptibility to cracking. Thus, improving the performance of concrete has become increasingly crucial [1–3]. To meet the diverse requirements of modern construction engineering, fiber-reinforced concrete (FRC) has been widely adopted. The incorporation of one or more types of fibers enables them to synergistically coordinate and complement each other, thereby endowing concrete with comprehensive performance enhancements, including improved crack resistance and toughness [4–5].

The interfacial adhesive layer between fibers and concrete is a weak zone within concrete. The existence of this adhesive layer prevents fibers from fully exerting their reinforcing and toughening effects on the concrete matrix. The characteristic properties of the adhesive layer are generally measured through fiber pull-out tests. Mao et al. [6] studied early crack resistance and microscopic mechanisms of basalt-polypropylene fiber-reinforced concrete via restrained ring tests. Results showed fiber addition markedly inhibits early shrinkage crack initiation and propagation; additionally, basalt and polypropylene fibers exert a favorable hybrid positive effect, effectively extending time to first cracking and reducing crack width. Further SEM analysis confirmed that fibers boost fiber-matrix adhesion via bridging and enhanced interfacial transition zone (ITZ) density, thereby significantly improving the concrete’s overall crack resistance and durability. Chen [7] studied the adhesion performance between basalt-polypropylene hybrid fiber-reinforced concrete and deformed steel bars via central pull-out tests, analyzing fiber content/aspect ratio effects on adhesion performance and evaluating rigid-flexible hybrid fiber toughening. Results showed hybrid fiber incorporation increased ultimate adhesion strength by 29%, improved the adhesion stress reduction trend, and enhanced adhesion failure ductility. But excessive fibers caused splitting failure and weakened adhesion performance. Hu [8] conducted experimental studies on different types of basalt fibers to explore the interfacial adhesion performance between basalt fibers and the concrete matrix. Using single-fiber pull-out tests, the fiber failure modes were identified, and the maximum pull-out load as well as the interfacial adhesion strength were quantified. The results demonstrated that fiber type and surface morphology exert a considerable influence on the pull-out response, with different fibers exhibiting distinct load-displacement characteristics. Moreover, increasing embedment depth and matrix strength can effectively enhance the fiber-matrix interfacial adhesion performance. Qin [9] investigated the interfacial adhesion performance between coarse polypropylene fibers and the cementitious matrix by conducting fiber pull-out tests, sulfate erosion tests, and dry-wet cycling experiments. In the pull-out tests, the influence of several factors, including curing age, loading rate, embedment depth, and fly ash content on interfacial adhesion performance was systematically examined. Huang [10] conducted tests on basalt-polypropylene fiber-reinforced concrete with different volume contents to investigate its mechanical properties, permeability, and pore structure. The results indicated that the splitting tensile strength of concrete increased with the incorporation of fibers, and basalt fibers exhibited a more pronounced reinforcing effect than polypropylene fibers. Both fiber types are capable of enhancing the impermeability of concrete, but adverse effects occurred when the content exceeded the optimal value.

With the development of commercial finite element software and meso-mechanical theory, the integration of experimental testing and numerical simulation has become an important approach for investigating the behavior of concrete materials. Compared with ordinary concrete, fiber-reinforced concrete exhibits a more complex internal stress distribution; therefore, numerical simulation can be used to reveal its mechanical behavior and failure mechanisms under loading. Luu [11] established multiple finite element models to simulate the interfacial performance between normal-strength concrete and ultra-high-performance fiber-reinforced concrete, adopting a surface-based cohesive model based on the traction–separation law. The model was validated in shear, tensile, and bending specimens, with the deviation between the model predictions and experimental data showing maximum values of 6.8% for ultimate load in shear tests, 15.9% for ultimate displacement in tensile tests, and 2.8% for ultimate displacement in bending tests. Muhammad [12] established finite element models to simulate the fracture behavior of steel fiber-reinforced concrete with different fiber contents, calculating fracture parameters such as fracture energy and fracture toughness. The compressive strength of the developed constitutive model was 28.50 MPa, which is very close to the experimental 28-day compressive strength of 28.79 MPa. Li [13] conducted pull-out tests and ABAQUS finite element numerical simulations on single coarse polypropylene fibers in cement mortar matrix, comparatively analyzing the effects of factors including fiber diameter, embedment length, water-binder ratio, and cement mortar matrix on interfacial adhesion properties, while verifying the accuracy of the numerical method. Based on micromechanics and the dynamic finite element method, Zhang et al [14] established a 2D dynamic numerical model of concrete double-wire pull-out. The study found that the matrix strength under dynamic load has a significant impact on the failure mode of the specimen. High-strength matrix mainly suffers from pull-out failure due to fiber interface debonding, while low-strength matrix causes joint pull-out failure of fiber and matrix, resulting in more severe matrix damage.

Recent studies have shown that the debonding behavior at fiber–concrete interfaces can involve complex mixed-mode fracture processes under three-dimensional stress states. In particular, the coupling between Mode I (normal separation) and Mode II (shear sliding) has been demonstrated to significantly influence the initiation and propagation of interfacial cracks. Several researchers have developed advanced 3D cohesive zone models to capture the mixed-mode debonding behavior of FRP–concrete interfaces. For example, Zhang [15] proposed a coupled mixed-mode cohesive zone formulation to evaluate interfacial debonding between FRP and concrete. Subsequently, Zhang [16] conducted a detailed three-dimensional numerical investigation of mixed-mode debonding mechanisms, highlighting the importance of circumferential stress states in the interfacial fracture process. A recent review by Huang [17] further summarized the current progress in cohesive zone modeling of FRP–concrete interface bond behavior.

Since Griffith [18] established linear elastic fracture mechanics in 1920, the study of damage and failure in engineering materials and structural components has remained a central focus and enduring challenge within the field of solid mechanics. Substantial advances in stress intensity factor theory, cohesive crack modeling, and the development of the J-integral have contributed to the progressive maturation of fracture mechanics theory. Kachanov [19] proposed a damage model for high-temperature creep of metals in 1958. It was not until the 1980s that crack band theory was formulated to address constitutive relationships characterized by strain-softening behavior, leading to substantial advancements in continuum damage mechanics. By integrating fracture mechanics and damage mechanics with numerical techniques such as the finite element method, researchers have developed both discontinuous and continuous approaches for damage and failure analysis. Consequently, damage and failure mechanics have progressively evolved into a significant branch of solid mechanics, playing a crucial role in structural safety assessment and optimal engineering design.

In recent years, the phase-field model (PFM) has emerged as a significant research focus in the field of fracture mechanics, owing to its exceptional capability in handling complex topological crack evolution. This approach can be traced back to the variational principle of fracture energy proposed by Francfort and Marigo [20] in 1998. This principle treats crack propagation as a process of minimizing the total potential energy of the system, fundamentally overcoming the limitations of classical fracture mechanics that require pre-defined crack paths. Subsequently, Miehe et al. [21] established the thermodynamic framework for phase-field fracture and introduced the strain energy spectral decomposition technique [22] to capture the asymmetric tension-compression failure behavior of quasi-brittle materials. These advancements marked the preliminary formation of the modern phase-field fracture theoretical system. However, the simulation results of classical AT1 and AT2 phase-field models exhibit extreme sensitivity to the length-scale parameter. Specifically, the predicted structural load-bearing capacity tends to increase abnormally as the length scale decreases, making it difficult for these models to provide an objective evaluation of the initial crack initiation point. Although some researchers have attempted to calibrate the length scale as an intrinsic material property related to the elastic modulus, tensile strength, and fracture energy, this approach is primarily applicable to purely brittle materials such as PMMA and ceramics [23]. For quasi-brittle materials like concrete, which exhibit significant softening characteristics, an excessively large length scale often leads to blurred crack paths and inaccurate predictions of the initiation load.

To address these deficiencies, Wu [24] conducted an in-depth investigation into the general forms of crack geometric functions and energy degradation functions, proposing the Unified Phase-Field Theory (UPFT), and then developed the Phase-Field Regularized Cohesive Zone Model (PF-CZM), specifically designed for quasi-brittle fracture. Compared to traditional AT1/AT2 models, PF-CZM offers significant physical advantages: its predictions become independent of the phase-field length scale within a reasonable range of values. This characteristic enables the model to more realistically simulate the entire process of concrete failure, from micro-crack initiation and macro-crack evolution to ultimate failure. Currently, PF-CZM has demonstrated high application value in research involving multiphase composites and complex fracture evolution [25], becoming a powerful tool for capturing the multi-modal cohesive fracture behavior of quasi-brittle materials.

To summarize, this paper established corresponding finite element models based on relevant fiber pull-out experiments. The bilinear cohesive zone model (CZM) was employed for the interfacial adhesive layer between basalt/polypropylene fibers and the concrete matrix, while the concrete PFM was adopted for the concrete matrix. Since the dominant failure mechanism in fiber pull-out problems is the progressive debonding along the fiber–matrix interface, the PF-CZM provides a more appropriate framework for capturing the interfacial softening behavior and crack propagation. This paper investigates the adhesion performance between fibers and concrete matrices by establishing numerical models of different fibers being pulled out of the matrix. The effects of embedment depth, fiber diameter, and interfacial properties on the pull-out mechanical behavior of the two types of fibers are analyzed. Combined with the PF-CZM, the damage evolution process is explored more intuitively, thus laying a valuable theoretical foundation for practical engineering applications.

## 2. Theoretical basis and governing equations

### 2.1 Cohesive zone model

**Fig 1:**
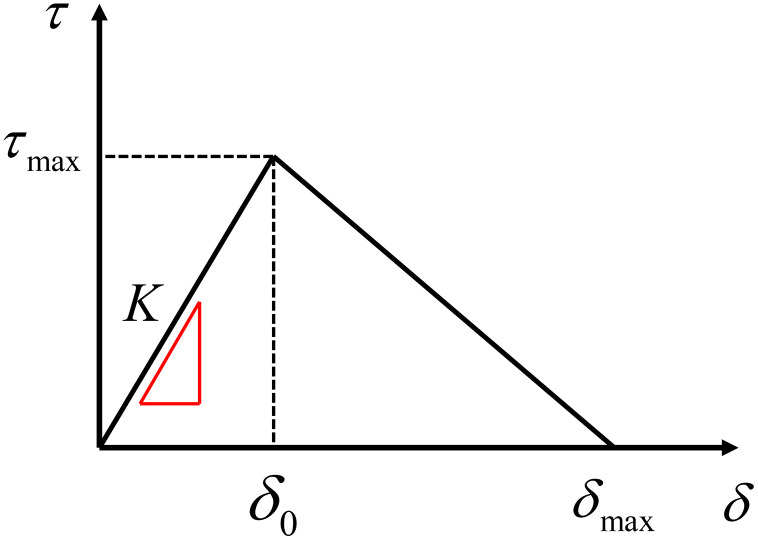
Constitutive curve of the bilinear CZM.

In this study, a layer of zero-thickness cohesive elements is inserted between the fiber and the concrete matrix to simulate the interfacial bonding behavior. Several types of cohesive zone models have been proposed in the literature, including bilinear, exponential, trapezoidal, and polynomial traction–separation relationships [26]. The first two models are suitable for analyzing brittle and quasi-brittle fractures, whereas the trapezoidal cohesive model is suitable for ductile fractures. The last one applies to various nonlinear, ductile fracture behaviors. Among these models, the CZM is widely used due to its simplicity and ability to effectively capture the key stages of interfacial damage evolution, including elastic response, damage initiation, and progressive softening. Therefore, the CZM is adopted in this study, and its constitutive relationship is illustrated in Fig 1.

$\tau_{max}$ is the normal adhesion strength, representing the maximum normal adhesion force that the interface can withstand before separation. $\delta_{max}$ is the normal failure displacement, representing the maximum normal separation distance at which the interface can still transmit adhesion force. When $\delta >\delta_{max}$, it indicates complete debonding of the interface, with the cohesive elements on both sides detaching from each other. The area τ δ enclosed by the curve and the horizontal axis is the interfacial fracture energy $G_{C}$; $K_{\text{0}}$ denotes the initial shear stiffness. As the three key parameters of the CZM, the interfacial fracture energy $G_{C}$, adhesion strength τ, and failure displacement δ have a relationship expressed as follows:


$$G_{C}=\frac{\text{1}}{\text{2}}\tau_{max}\delta_{max}$$ (1)

Bilinear cohesive elements have their specific criteria for determining the process from the initiation of damage to its gradual development and evolution. The damage initiation criterion serves to identify the onset of damage and subsequent stiffness degradation. In this paper, the maximum nominal stress criterion was selected as the initiation criterion, and the initiation of damage satisfies the following conditions:


$$max\{\frac{\langle \tau_{n}\rangle }{\overset{\text{0}}{\underset{n}{\tau }}},\frac{\tau_{t}}{\overset{\text{0}}{\underset{t}{\tau }}}\}=1$$ (2)

Where $\overset{\text{0}}{\underset{n}{\tau }}$ and $\overset{\text{0}}{\underset{t}{\tau }}$ respectively represent the maximum stress values that can be borne in the normal direction and shear direction.

After the initial damage occurs, damage evolution ensues. The damage evolution criterion in the CZM is used to express the relationship between the separation displacement and the traction force, and the degradation of its mechanical properties is characterized by stiffness degradation. The damage evolution can be expressed as:


$$D=\left\{ \begin{array}{c} @l@0,\;\;\;\;\delta \leq \delta_{\text{0}}\\ \frac{\delta_{max}(\delta -\delta_{\text{0}})}{\delta (\delta_{max}-\delta_{\text{0}})},\;\delta >\delta_{\text{0}} \end{array}\right.$$ (3)

Stiffness degradation is represented by D. Its value range is 0–1. When D=0, it indicates that no damage has occurred; when D=1, it indicates complete damage. After damage occurs, the stiffness of the cohesive element degrades to:


$$K=\left(1-D\right)K_{\text{0}}$$ (4)

The total interfacial response is decomposed into a cohesive part and a frictional part. The cohesive part follows the traction-separation law (Eq. 3), while the frictional part is governed by the relationship $\tau_{fric}=\mu \cdot \sigma_{n}$, where $\sigma_{n}$ is the contact pressure. This dual-mechanism ensures that the residual load-bearing capacity is maintained after the chemical bond is exhausted.

### 2.2 Unified phase-field theory

Existing studies have shown that fiber failure modes within the concrete matrix are not limited to interfacial failure; the damage and failure of the concrete matrix itself are the primary failure modes resulting in fiber pull-out. In this paper, a concrete phase-field damage model based on the UPFT was adopted. The basic concepts and main equations of the UPFT are briefly presented herein [27, 28].

**Fig 2:**
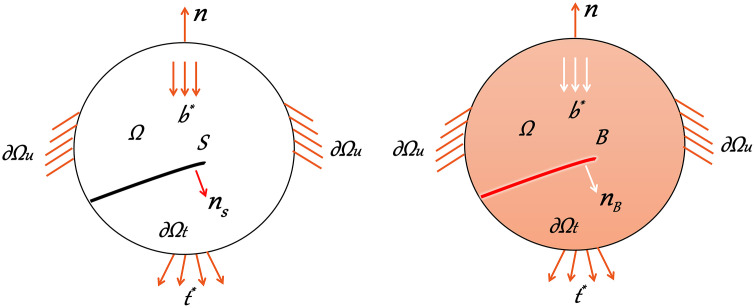
Fracture diagram of PFM. (a) Sharp crack; (b) Localized representation.


As shown in Fig 2 (a), the cracked solid specimen is denoted as $\Omega \subset {\mathbb{R}}^{n_{dim}}$ ($n_{dim}$ = 1,2,3), and b* represents the body force acting on the solid. The displacement field of this solid is denoted as u(x), and the strain field of the solid is denoted as $\varepsilon \left(x\right):=\nabla^{\text{sym}}u\left(x\right)$. The boundary of the solid is divided into two parts, $\partial \Omega_{\text{u}}$ and $\partial \Omega_{\text{t}}$, which are applied on two non-intersecting boundaries respectively—meaning they satisfy $\partial \Omega_{\text{u}}⋂\partial \Omega_{\text{t}}=\emptyset$ and $\partial \Omega_{\text{u}}⋃\partial \Omega_{\text{t}}=\partial \Omega$. The displacement boundary condition $u^{*}\left(x\right)$ is applied at $x\in \partial \Omega_{\text{u}}$, and the external force boundary condition $t^{*}\left(x\right)$ is applied at $x\in \partial \Omega_{\text{t}}$. Then, the admissible space $U_{\text{u}}$ of this displacement field can be expressed as:

$$U_{\text{u}}:=\{u|u\left(x\right)=u^{*}\;\forall x\in \partial \Omega_{\text{u}}\}$$ (5)

When a solid specimen is subjected to force, once the requirements of the cracking criterion are met, sharp cracks form inside the solid, as shown in Fig 2 (b). In the PFM, sharp cracks 𝒮 are regularized over a localization band ℬ⊆Ω. A continuous damage field d(x) is present within this localization band, whose outer boundary is denoted as ∂B; the region outside this band remains undamaged. The space $U_{\text{d}}$ of the damage phase-field can be expressed as:


$$U_{\text{d}}:=\{d|d\left(x\right)\in \left[0,1\right]\text{,}\dot{d}\left(x\right)\geq 0\;\forall x\in ℬ;d\left(x\right)=1\;\forall x\in 𝒮\}$$ (6)

Where the continuous damage field $\dot{d}\left(x\right)\geq 0$ indicates that the damage is irreversible.

#### 2.2.1 Constitutive relation.

According to the first and second laws of thermodynamics, the energy release rate inequality for any admissible deformation process of the solid can be derived as follows:


$$\dot{𝒟}=\int_{\Omega }\left(\sigma :\dot{\varepsilon }-\dot{\psi }\right)\text{d}V\geq 0$$ (7)

ε represents the strain field, σ represents its corresponding stress field, and ψ represents the local free energy density of the solid. When considering the case of isotropy, ψ can be expressed as the following general form:


$$\psi \left(\varepsilon ,d\right)=\omega \left(d\right)\psi_{\text{0}}\left(\varepsilon \right)$$ (8)

$$\psi_{\text{0}}\left(\varepsilon \right)=\frac{\text{1}}{\text{2}}\varepsilon :{𝔼}_{\text{0}}:\varepsilon =\frac{\text{1}}{\text{2}}\overset{―}{\sigma }:{\mathbb{C}}_{\text{0}}:\overset{―}{\sigma }$$ (9)

$\psi_{\text{0}}\left(\varepsilon \right)$ is the initial strain energy density; ω(d) is the energy degradation function; ${𝔼}_{\text{0}}$ is the elastic modulus of the material; ${\mathbb{C}}_{\text{0}}$ is the material compliance; $\bar{\sigma }=E_{\text{0}}\cdot \varepsilon$ is the effective stress tensor.

The strain energy density is decomposed into tensile and compressive components by strain spectral decomposition. Only the tensile strain energy density $\overset{―}{Y}$ after processing with the energy degradation function constitutes the effective damage driving force Y for phase-field evolution:


$$Y:=\frac{\partial }{\partial d}\psi \left(\varepsilon ,d\right)=-\omega^{\prime }\left(d\right)\overset{―}{Y}$$ (10)

Where $\overset{―}{Y}=\partial \psi /\partial \omega$ represents the effective damage driving force.

Combined with the above formula, the constitutive relation can be obtained:


$$\left\{ \begin{array}{c} @l@\sigma :=\frac{\partial \psi }{\partial \varepsilon }=\omega \left(d\right){𝔼}_{\text{0}}:\varepsilon \\ Y:=-\frac{\partial \psi }{\partial d}=-\frac{\text{1}}{\text{2}}\omega^{\prime }\left(d\right)\varepsilon :{𝔼}_{\text{0}}:\varepsilon \end{array}\right.$$ (11)

#### 2.2.2 Law of damage evolution.

Building upon Griffith theory and classical PFM, sharp cracks can be regularized and characterized via mathematical formulations within localization bands ℬ.


$$A_{S}=\int_{S}dA\approx \int_{B}\gamma \left(d\right)dV=A_{d}\left(d\right)$$ (12)

γ(d) denotes the crack surface density, and its expression is as follows:


$$\gamma \left(d\right)=\frac{\text{1}}{c_{\text{0}}}\left[\frac{\text{1}}{b}\alpha \left(d\right)+b{\left|\nabla d\right|}^{\text{2}}\right],\;c_{\text{0}}=4\int_{\text{0}}^{\text{1}}\sqrt{\alpha \left(\beta \right)}\text{d}\beta$$ (13)

b is a length scale parameter that governs the width of the localization band ℬ, serving as a pivotal parameter in the UPFT. The damage distribution within the localization band ℬ is dictated by the geometric crack function α(d)∈[0,1].

Furthermore, based on the principle of conservation of energy, the UPFT proposes that there exists the following relationship between the damage evolution within localization bands and the original sharp cracks:


$$\dot{D}=\int_{B}Y\dot{d}\text{d}V=G_{\text{f}}\int_{B}\dot{\gamma }\left(d\right)\text{d}V\approx G_{\text{f}}{\dot{A}}_{S}\geq 0$$ (14)

$G_{\text{f}}$ is the fracture energy. Building upon damage irreversibility $\text{(}\dot{\text{d}}\left(x\right)\geq 0)$ and Gauss’s divergence theorem, the damage evolution criterion and its corresponding Neumann-type boundary conditions can be derived as follows:


$$\left\{ \begin{array}{c} @l@g\left(Y,d\right):=Y-G_{\text{f}}\delta_{\text{d}}\gamma \leq 0\\ \nabla d\cdot n_{ℬ}=0\;\;on\;\partial ℬ \end{array}\right.$$ (15)

${\text{n}}_{ℬ}$ represents the outward normal direction of the boundary ∂ℬ; and $\delta_{d}\gamma$ denotes the derivative of the crack surface density function with respect to damage.

#### 2.2.3 Phase-field regularized cohesive zone model.

The geometric crack function α(d) and energetic degradation function ω(d) are two core characteristic functions in the PFM. Jian-Ying Wu integrated the PFM and CZM, and thereby explicitly recommended practical values for α(d) and ω(d). Based on these recommendations, he proposed a phase-field damage model for concrete (PF-CZM), with the specific recommended forms shown as follows:


$$\alpha \left(d\right)=\xi d+\left(1-\xi \right)d^{\text{2}}$$ (16)

$$\;\omega (d)=\frac{(\text{1}-d{)}^{p}}{\left(\text{1}-d{)}^{p}+Q(d\right)}\;Q(d)=a_{\text{1}}d+a_{\text{1}}a_{\text{2}}d^{\text{2}}+a_{\text{1}}a_{\text{2}}a_{\text{3}}d^{\text{3}}\;p\geq \text{2}$$ (17)

The coefficients $a_{\text{1}}\;,\;a_{\text{2}}\;,\;a_{\text{3}}$ are derived from the material and as follows:


$$a_{\text{1}}=\frac{\text{4}}{\pi b}\cdot \frac{E_{\text{0}}G_{\text{f}}}{\overset{\text{2}}{\underset{t}{f}}}$$ (18)

$$a_{\text{2}}=2{\left(-2\frac{G_{f}}{\overset{\text{2}}{\underset{t}{f}}}\cdot k_{\text{0}}\right)}^{\frac{\text{2}}{\text{3}}}-\left(p+\frac{\text{1}}{\text{2}}\right)$$ (19)

$$a_{\text{3}}=\left\{ \begin{array}{c} @l@0,\text{ p > 2}\\ \frac{\text{1}}{a_{\text{2}}}\left[\frac{\text{1}}{\text{2}}{\left(\frac{f_{\text{t}}{\hat{u}}_{\text{u}}}{2G_{\text{f}}}\right)}^{\text{2}}-\left(1+a_{\text{2}}\right)\right],\text{p = 2} \end{array}\right.$$ (20)

$k_{\text{0}}$ represents the initial stiffness, b denotes the length scale parameter, and $u_{u}$ stands for the final cracking displacement.

When specific values are assigned to p and $a_{i}(i=1,2,3)$, the concrete softening law can be fitted. This study adopts the widely used Cornelissen softening law for concrete, which is calibrated against concrete experimental data to enhance its adaptability to concrete. Under this condition, p=2, $a_{\text{2}}\text{=1.3968}$, $a_{\text{3}}\text{=0.6567}$.

### 2.3 Numerical implementation

Based on the constitutive relation and law of damage evolution, the governing terms in strong form can be expressed as


$$\left\{ \begin{array}{c} @l@\nabla \cdot \sigma \text{+}b^{*}=\frac{\partial \psi }{\partial \varepsilon }=\text{0\textbackslash{}\textbackslash{}\textbackslash{}\textbackslash{}\textbackslash{}\textbackslash{}\textbackslash{}\textbackslash{},\textbackslash{}in }\Omega \\ -Y+G_{f}\delta_{d}\gamma \geq 0,\forall \dot{d}\geq 0\text{ in}\;ℬ \end{array}\right.$$ (21)

As can be seen, the UPFT starts from thermodynamics and transforms the complex structural damage problem and fracture mechanics problem into two governing equations; the upper equation is used to solve the displacement field problem, and the other is used to solve the damage phase-field problem. Its Neumann (second type) boundary condition expression is:


$$\left\{ \begin{array}{c} @l@\sigma \cdot n=t^{*}\;\;\text{ on}\partial \Omega_{t}\\ \nabla d\cdot n_{B}=0\text{ on}\partial ℬ \end{array}\right.$$ (22)

In the two-dimensional (2D) case, the computational domain Ω of the deformable solid can be discretized into a grid h, where the superscript h indicates the grid size of the finite element domain. To ensure the accuracy of the calculation results, the grid size should be much smaller than the crack scale b, typically taking 1/5 h ≤ b.

The numerical implementation of the PF-CZM was carried out in ABAQUS through a user-defined material subroutine (UMAT). The UMAT was developed based on the interface provided by ABAQUS and coded using the Fortran programming language. The above governing equations are solved using the overall BFGS quasi-Newton iterative algorithm. Through this user-defined subroutine, the PF-CZM for concrete was incorporated into the finite element framework, enabling the simulation of crack initiation, propagation, and the progressive damage process of the material.

## 3. Numerical model establishment and validation for basalt and polypropylene fiber pull-out

### 3.1 Description of reference experiments

The experimental data used for validating the numerical model were obtained from previously published fiber pull-out tests. Two different types of fibers, namely basalt fiber and polypropylene fiber, were considered in this study.

For the basalt fiber pull-out test, the experimental data were taken from Hu [8]. The experiment was conducted using a cylindrical model with a diameter of 100 mm and a height of 200 mm. The pull-out tests were conducted using an AG-X-plus universal testing machine. Loading was performed in a quasi-static manner with displacement control, and the loading speed was 0.025 mm/s. The concrete matrix was prepared using ordinary Portland cement, natural sand, and crushed stone aggregates. The concrete grade was C40, with a water–cement ratio of approximately 0.42. The mix proportions were: water 168 kg, cement 400 kg, sand 567 kg, and coarse aggregate 1261 kg.

For the polypropylene fiber pull-out test, the experimental data were obtained from Qin [9]. The experiment used a square specimen with a side length of 100 mm, where the test procedure followed the recommendations of CECS13:2009 (Test Method for Steel Fiber Reinforced Concrete). The pull-out tests were performed using a 5 kN tensile testing machine, with a force accuracy of 0.3%. The main raw materials included Portland cement, fine aggregate, fly ash, and water. The cement used was ordinary Portland cement. The mix proportion corresponded to a water–cement ratio of 0.40, with a mass ratio of water: cement: fly ash: sand = 1: 2.5: 0.25: 3.3.

After casting, the specimens were demolded after 24 h and then cured in a standard curing room at a temperature of 20 ± 2 °C and a relative humidity of over 95% for 28 days before testing. These experimental results were used to validate the numerical model developed in this study.

**Fig 3:**
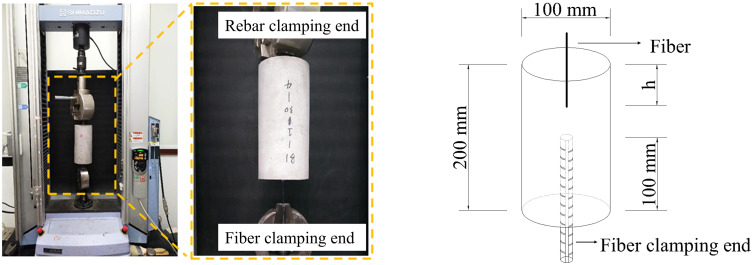
The schematic diagram of the basalt fiber Pull-out test: (a) Testing instruments; (b) Schematic diagram of fiber pull-out specimen.

**Fig 4:**
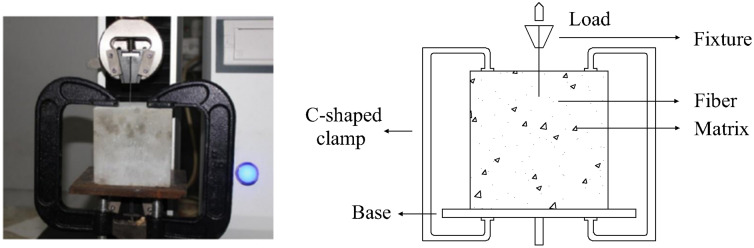
The schematic diagram of the polypropylene fiber Pull-out test: (a) Testing instruments; (b) Device schematic diagram.

The experimental setup is schematically illustrated in Figs 3 and 4. The functional relationship between the measured pull-out load and the corresponding displacement characterizes fiber adhesion performance. This research examined the influence of factors such as varying fiber embedment depths and diameters on the interfacial adhesion performance of the fibers.

### 3.2 Establishment of finite element model

To facilitate mesh generation and numerical calculation, the cylindrical specimen is simplified into a cuboid model with equal height and width. For the basalt fiber pull-out test, the concrete matrix measures 200 mm × 100 mm, and the fiber diameter is 0.90 mm. For the polypropylene fiber pull-out specimen, the concrete matrix measures 100 mm × 100 mm, and the fiber diameter is 0.90 mm. In the present study, the length scale parameter *b* of the PF-CZM was set to 1 mm, which controls the width of the diffused crack zone. The finite element mesh size in the region surrounding the fiber and the interface was refined to 0.25 mm, which ensures an accurate representation of crack propagation. Both numerical models use the same mesh size. The numerical results showed negligible differences when further refining the mesh (0.2 mm, 0.1 mm), indicating that the adopted mesh density is sufficient to ensure mesh-independent results.

In the numerical simulation, a 2D plane-stress model was adopted to represent the fiber pull-out specimen. The fiber is subjected to a vertical downward concentrated force and restrained from vertical displacement on the upper side of the concrete matrix, and the bottom surface of the concrete matrix is fixed in the loading direction. The lateral surfaces remained free to allow for natural Poisson-induced deformation. Although the specimen has a certain thickness, the deformation in the out-of-plane direction is not significantly constrained during the pull-out process. Preliminary sensitivity analysis confirmed that for the large matrix-to-fiber volume ratio used in this study, the presence or absence of lateral constraints has a negligible effect (less than 1%) on the interfacial stress transfer and peak pull-out force.

Therefore, the plane-stress assumption is considered reasonable for capturing the interfacial debonding behavior between the fiber and the surrounding concrete matrix. In addition, the use of a 2D model greatly reduces computational cost while maintaining sufficient accuracy for the parametric analysis conducted in this study. Similar 2D simplifications have been widely adopted in previous studies on fiber pull-out simulations.

For the numerical calculation of this finite element model, firstly, the fiber and concrete matrix parts were established. Subsequently, different element sets were assigned to each part and then assembled into a unified model. The concrete matrix part adopts the PF-CZM, with an ABAQUS CPS4T element selected. For the basalt fiber component, a linear model was adopted, and the CPS4 element was chosen. Then, zero-thickness cohesive elements were embedded between these two sets to accurately represent the interfacial adhesion between the fiber and the concrete matrix. This cohesive element utilizes the bilinear CZM, with the COH2D4 element selected.

The material parameters of basalt fiber, polypropylene fiber, and the concrete matrix were obtained from refs. [10–12], and these parameters are shown in Table 1. During the finite element simulation, two parameters of the adhesion strength and fracture energy were initially unknown.

**Table 1:** Material parameters.

Material	Elasticity Modulus E (GPa)	Poisson’s Ratio υ	Tensile Strength $f_{t}$ (MPa)	Fracture energy $G_{c}$ (N/mm)
Concrete matrix	25	0.173	2.9	0.15
Polypropylene fiber	3.7	0.3	—	—
Basalt fiber	85	0.25	—	—

In this study, the interfacial parameters were determined through a systematic calibration-validation procedure. Specifically, the experimental data from the 10 mm embedment depth case were used as the calibration benchmark to identify the optimal interfacial properties. Subsequently, these identified parameters were fixed and applied to simulate the 8 mm and 12 mm embedment cases without further adjustment. This approach ensures that the model’s predictive capability is rigorously tested against independent experimental datasets. The interface parameters are shown in Table 2.

**Table 2:** Cohesive interfacial parameters used in the numerical simulation.

Interfacial tensile strength $f_{t}$ (MPa)	Interfacial shear strength $f_{t}$ (MPa)	Fracture energy $G_{c}$ (N/mm)	Elastic stiffness (MPa/mm)
3.0	3.0	0.08	1 × 10^6^

### 3.3 Verification and analysis of the model

**Fig 5:**
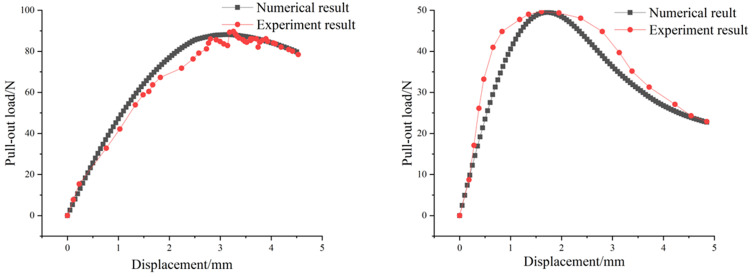
Peak pull-out load and displacement of different fibers with different embedment depths: (a) Basalt fiber; (b) Polypropylene fiber.

Fig 5 presents comparisons between the load-displacement curves obtained from numerical simulations and the experimental results of basalt fiber and polypropylene fiber pull-out tests reported in ref [11–12]. The vertical axis represents the vertical upward pull-out force applied to the two types of fibers, while the horizontal axis denotes the relative displacement between the fibers and the concrete matrix. For the basalt fiber, when the adhesion strength τ of the cohesive element was set to 0.2 MPa, and the fracture energy $G_{C}$ to 0.3 N/mm, the peak pull-out load and the corresponding displacement obtained from the simulation were 88.1124 N and 3.1 mm, respectively. The experimental results were 89.27 N and 3.25 mm, with errors of 1.3% and 4.6%, respectively. For the polypropylene fiber, when the adhesion strength of the cohesive element τ was set to 0.1 MPa, and the fracture energy $G_{C}$ to 0.25 N/mm, the simulated peak pull-out force and the corresponding displacement were 49.49 N and 1.7 mm, while the experimental results are 49.67 N and 1.61 mm. The errors were 0.4% and 5.3%, respectively. Overall, the numerical simulation results are in good agreement with the experimental results.

As shown in Fig 5, the simulation results of this study are in good agreement with the experimental results. The entire fiber pull-out process can be roughly divided into four distinct stages:

Stage I (Initial Tension Stage): When the fiber was under tension, the load and displacement increased linearly. During this period, the fiber and concrete matrix are in an elastic bond state, with no interface slippage, damage, or cracks. Stage II (Progressive Debonding Stage): As the applied load increased, the shear stress between the fiber and the concrete matrix reached the interfacial bond strength, and debonding began to occur near the loaded end of the fiber. The load and displacement increase non-linearly until the tensile force reaches its maximum value. In this stage, microcracks first initiate at the loading end and propagate steadily along the fiber-matrix interface. Stage III (Complete Debonding Stage): With further increases in displacement, the bonded interface degrades until it completely fails. The fiber and matrix achieve complete debonding, and the mechanical interlocking effect between the fiber and the interface gradually weakens. The interfacial cracks completely penetrate along the buried length direction. Stage IV (Friction-Dominated Stage): The load is entirely borne by interfacial frictional resistance. As the fiber continues to be pulled out, the frictional force gradually decreases, and the pull-out load decreases accordingly. At this point, the interface cracks no longer expand; the only manifestation is that the crack width continues to increase.

**Fig 6:**
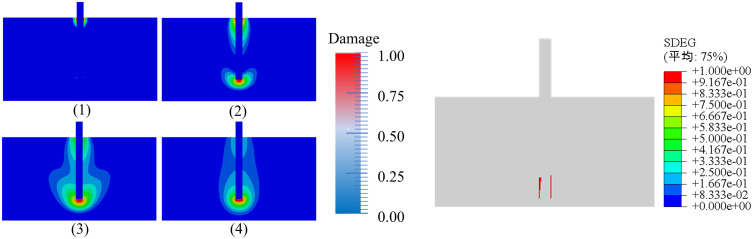
Diagram of damage distribution: (a) Matrix damage cloud map; (b) Interface damage diagram.

The evolution process of local damage from 0 to 1 can be observed in Fig 6a(1–4), which represents displacements of 0.1, 1.0, 2.0, and 4.5, respectively. The concrete matrix at the bottom of the fiber was damaged as the fiber was pulled out, relative slip occurred between the fiber and the interface, and the fiber was gradually pulled out. Therefore, whether from a macroscopic perspective, mesoscopic numerical simulations, or quantitative comparison, the concrete phase-field damage model accurately reflects real physical crack behavior and is suitable for studying fiber pull-out. Based on this analysis, it can be concluded that the finite element model established in this study can effectively reproduce the entire pull-out process of both types of fibers from the concrete matrix, demonstrating a certain degree of accuracy and reliability.

It should be noted that fiber rupture was not observed in the pull-out experiments. The dominant failure mode was interfacial debonding followed by fiber pull-out. Therefore, the fiber was modeled as a linear elastic material without considering fiber damage.

## 4. Effects of different factors on the pull-out mechanical properties of basalt fibers and polypropylene fibers

### 4.1 Effect of fiber embedment depth

**Fig 7:**
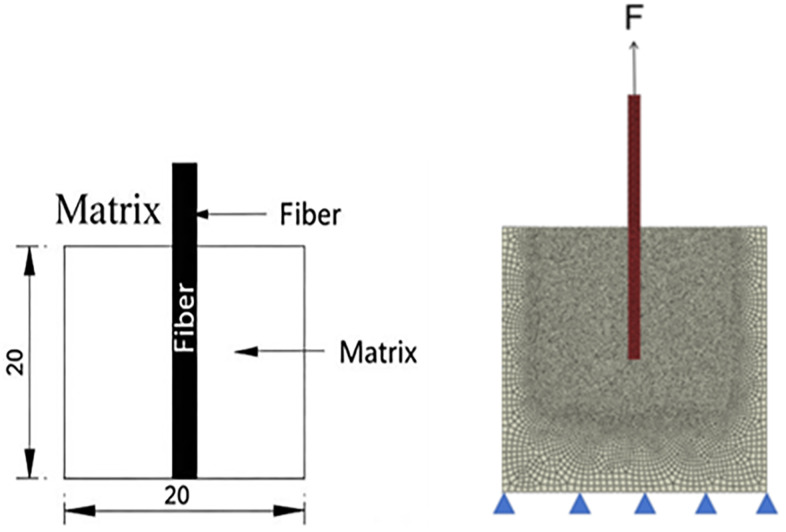
Geometric conditions and boundary conditions of fiber pull-out model: (a) Geometric conditions; (b) Boundary conditions.

To conduct the effects of various factors on the pull-out mechanical properties of basalt fibers and polypropylene fibers while ensuring computational efficiency, this study established a unified finite element model, as shown in Fig 7(a). The model features a concrete matrix with dimensions of 20 mm × 20 mm and a fiber diameter of 0.90 mm, with detailed structural parameters, boundary conditions, and loading methods illustrated in Fig 7(b). Using this model, vertical pull-out simulation analyses were performed, focusing on investigating the influence of fiber embedment depth, fiber diameter, and interfacial properties on the pull-out mechanical behavior of the two fiber types.

**Fig 8:**
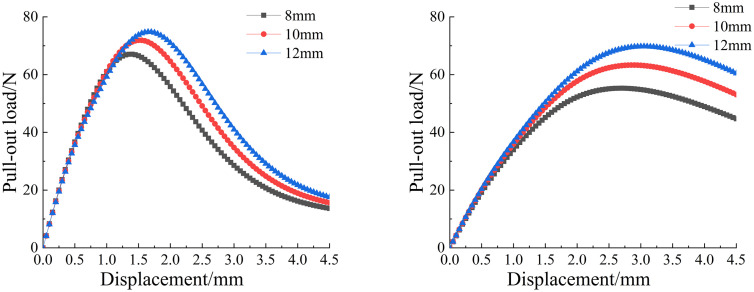
Pull-out load–displacement curve of different fibers with different embedment depths: (a) Basalt fiber; (b) Polypropylene fiber.

**Fig 9:**
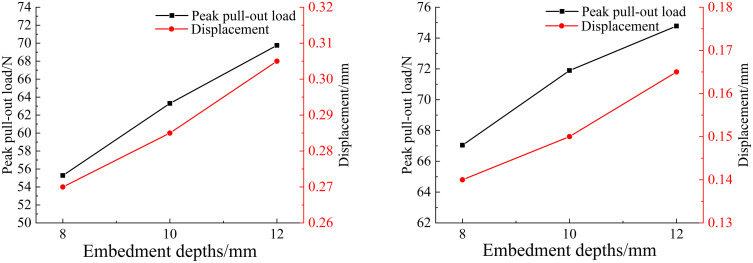
Peak pull-out load and displacement of different fibers with different embedment depths: (a) Basalt fiber; (b) Polypropylene fiber.

To investigate the effect of different embedment depths on the pull-out mechanical properties of the two fiber types, finite element models with embedment depths of 8 mm, 10 mm, and 12 mm were established to simulate the pull-out process, as shown in Fig 8. All other parameters were kept consistent with the previously described model configuration. Fig 9 shows the load-displacement curves of basalt fibers and polypropylene fibers under different embedment depths.

The analysis results showed that the distribution patterns of the pull-out load-displacement curves for the three groups are generally consistent, with the ascending segments partially overlapping. This indicates that for the two different types of fibers, the embedment depth has no effect before debonding occurs. As the embedment depth increases, the contact area between the two types of fibers and the concrete matrix increases; thus, the peak pull-out load also increases accordingly. For basalt fibers with different embedment depths, the pull-out load-bearing capacity of those with 10 mm and 12 mm embedment depths was 14.5% and 26.2% higher than that of the 8 mm reference depth, respectively. For polypropylene fibers, the corresponding increases were 7.2% and 11.5% for the 10 mm and 12 mm embedment depths compared to the 8 mm depth. These simulation results indicate that as the fiber embedment depth increases, the pull-out bearing capacity of both types of fibers increases, and the displacement corresponding to the peak pull-out load also increases slightly. Therefore, the embedment depth exerts a significant influence on the pull-out mechanical properties of both basalt and polypropylene fibers.

### 4.2 Effect of fiber diameter

**Fig 10:**
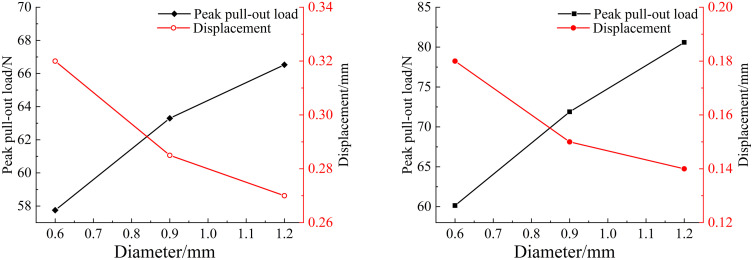
Variation of peak pull-out load and displacement of different fibers with different diameters: (a) Basalt fiber; (b) Polypropylene fiber.

Fiber diameter is also a crucial parameter. To investigate the effects of two fiber diameters on the pull-out mechanical properties, finite element models with embedment depths of 0.6 mm, 0.9 mm, and 1.2 mm were established, with a consistent embedding depth of 10 mm. The pull-out processes of the two fibers were simulated, and the peak pull-out load and their corresponding displacement are presented in Fig 10, respectively.

As can be seen from the simulation results, when the embedding depths of the two types of fibers are the same, the larger the diameter of the fiber, the greater the tensile force required for its pull-out. For basalt fibers, when the diameters were 0.9 mm and 1.2 mm, the peak loads increased by 9.6% and 15.2% respectively compared with that at a diameter of 0.6 mm; for polypropylene fibers, when the diameters were 0.9 mm and 1.2 mm, the peak loads increased by 19.5% and 34% respectively compared with that at a diameter of 0.6 mm. This also indicates that thicker basalt fibers and polypropylene fibers can provide superior reinforcement for concrete. However, it does not imply the simplistic rule of “the thicker the fiber, the better”; excessively thick fibers are detrimental to structural load-bearing capacity and significantly compromise concrete’s ductility and toughness.

### 4.3 Influence of interfacial properties

Existing fiber pull-out tests have shown that there are primarily two typical failure modes at the fiber-matrix interface: one involves interfacial failure between fibers and concrete, which results in fiber pull-out; the other occurs when interfacial adhesion strength of the fiber exceeds the tensile strength of the concrete matrix, causing failure and spalling of the concrete portion adhered to the fibers.

Building on these two failure modes, this study focuses on investigating the effects of the adhesion strength of cohesive elements within the adhesive layer and the tensile strength of the concrete matrix on the pull-out mechanical properties of the two fiber types.

(1) Effect of adhesion strength τ

**Fig 11:**
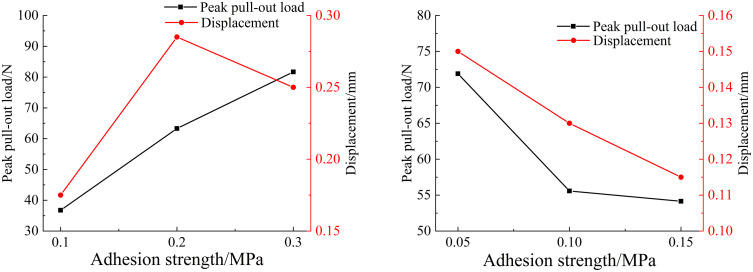
Variation of peak pull-out load and displacement of different fibers with interfacial adhesion strength: (a) Basalt fiber; (b) Polypropylene fiber.

When analyzing the influence of interfacial adhesion strength τ on the pull-out mechanical properties of basalt fibers, $f_{t}$ = 3N/mm, and three different values of $\tau_{max}$ = 0.1 MPa, 0.2 MPa, and 0.3 MPa, are selected, respectively, while other parameters remained unchanged. The peak pull-out load for basalt fiber obtained through numerical simulation is shown in Fig 11(a).

Similarly, for the analysis of polypropylene fibers, $f_{t}$ = 2.5N/mm, and chose three different values of $\tau_{max}$ = 0.05 MPa, 0.1 MPa, and 0.15 MPa, with other parameters kept unchanged. The peak tensile force for polypropylene fiber pull-out obtained via numerical simulation is presented in Fig 11(b).

The results indicate that the greater the adhesion strength of the interfacial layer of basalt fibers, the higher the peak pull-out force required to extract the fibers, and the better the fiber reinforcement effect. Specifically, when the interfacial adhesion strength $\tau_{max}$ was set at 0.2 MPa and 0.3 MPa, the pull-out bearing capacity of basalt fibers increased by 72% to 122% compared with that at 0.1 MPa. This confirms that the peak pull-out force of basalt fibers rises significantly as the interfacial layer adhesion strength increases. In contrast, the peak pull-out load of polypropylene fibers exhibits a decreasing trend with increasing interfacial adhesion strength. When the interfacial adhesion strength $\tau_{max}$ was set at 0.1 MPa and 0.15 MPa, the pull-out bearing capacity of polypropylene fibers decreased by 22.7% to 24.8% compared with that at 0.05 MPa.

This difference can be attributed to the distinct mechanical properties of the two types of fibers and their interaction with the surrounding concrete matrix. Basalt fibers possess a relatively high elastic modulus and tensile strength, which allows them to effectively transfer and sustain higher stresses when the interfacial adhesion strength increases. As a result, stronger interfacial bonding enhances the stress transfer efficiency between the fiber and the concrete matrix, leading to an increase in the peak pull-out load.

In contrast, polypropylene fibers have a much lower elastic modulus, and the load transfer at the interface is more sensitive to the interfacial traction–separation behavior. As the interfacial adhesion strength increases, the stress distribution along the interface changes and the debonding process may initiate earlier, which can reduce the peak pull-out load observed in the numerical results.

(2) Effect of tensile strength $f_{t}$

The tensile strength of the concrete matrix also exerts a significant influence on the peak pull-out load. For this analysis, the interfacial adhesion strength τ was fixed with 0.3 MPa for basalt fibers and 0.05 MPa for polypropylene fibers. Then three different groups of tensile strength values $f_{t}$ as 2 MPa, 2.5 MPa, and 3 MPa for both basalt fibers and polypropylene fibers, while all other parameters remained unchanged.

**Fig 12:**
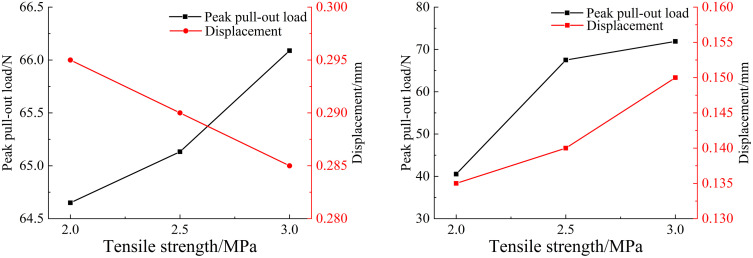
Variation of peak pull-out load and displacement of different fibers with tensile strength: (a) Basalt fiber; (b) Polypropylene fiber.

As shown in Fig 12, the results showed that when the tensile strength of basalt fiber was set to 2.5 MPa and 3 MPa, its corresponding peak pull-out load increased by 0.8% and 2.3% respectively, compared with that when the tensile strength was 2 MPa. With the increase in tensile strength, the peak pull-out load of basalt fiber increases slightly with a negligible growth margin. When the tensile strength of polypropylene fiber was 2.5 MPa and 3 MPa, its peak pull-out load increased by 66.7% and 77.5% respectively, compared with that when the tensile strength was 2 MPa. By comparing the two types of fibers, it has been observed that the enhancement of tensile strength in polypropylene fiber exerts a more significant influence on the increase in peak load than that of basalt fiber.

## 5. Conclusion

This study provides theoretical guidance for the design of fiber-reinforced concrete with improved interfacial performance. Based on the PF-CZM, this paper establishes finite element numerical model for the single-fiber pull-out of basalt fiber and polypropylene fiber. By comparing the model results with the experimental results, the reliability of the numerical model is verified. On this basis, this paper calculates and analyzes the influence of different factors on the interfacial adhesion performance between fibers and concrete matrix, and the following conclusions are as follows:

(1) The PF-CZM is used to simulate the pull-out of basalt fiber and polypropylene fiber. The obtained experimental results are in good agreement with the simulation results, which indicates that the application of the PF-CZM for the finite element simulation of basalt fiber and polypropylene fiber possesses a significant degree of reliability.
(2) Exploring factors affecting the pull-out mechanical properties of basalt and polypropylene fibers, results show that basalt fiber’s pull-out bearing capacity at 10 mm and 12 mm embedment depths is 14.5% and 26.2% higher, respectively, than the 8 mm reference; while polypropylene fibers’ increases by 7.2% and 11.5% at the same embedment depths vs. 8 mm. At the same embedment depth, larger diameters of both fibers enhance their pull-out bearing capacity. In practical engineering, these factors can be appropriately enhanced to improve the mechanical properties of fiber-reinforced concrete.
(3) By analyzing the effects of interfacial parameters on the pull-out behavior of basalt and polypropylene fibers, it is found that interfacial adhesion strength governs the mechanical response of basalt fibers, whereas matrix tensile strength plays a more dominant role for polypropylene fibers. When the adhesion strength increases from 0.1 MPa to 0.2–0.3 MPa, the pull-out capacity of basalt fibers rises by 72%−122%. Similarly, when the tensile strength increases from 2 MPa to 2.5–3 MPa, the pull-out capacity of polypropylene fibers improves by 66.7%−77.5%. Therefore, the reinforcing effect of fibers in concrete could be improved by means of fiber modification or fiber twisting.

The primary objective of this study is to investigate the interfacial bonding behavior and the influence of key parameters under single-fiber pull-out scenarios. To achieve this goal with reasonable computational efficiency, this paper only performs numerical simulations on 2D finite element models of basalt fiber or polypropylene fiber reinforced concrete. Since the pull-out configuration is inherently axisymmetric, the fibers are vertically embedded in the concrete matrix and bear axial loads. In this case, the PF-CZM can still effectively capture the main mechanical responses and crack propagation behavior, significantly reducing computational costs while preserving the basic characteristics of the pull-out process. 3D finite element models can more accurately describe the circumferential stress field and hybrid fracture mechanisms, reflecting the fiber pull-out test more realistically and comprehensively. Therefore, future research can establish 3D mesoscopic finite element models under various conditions based on the PFM.

## Supporting information

S1 File (XLSX)
